# Hohes Interaktionspotenzial volldigitaler Lehrveranstaltungen mit Breakout-Sessions – Ergebnisse einer Pilotstudie

**DOI:** 10.1007/s00106-023-01407-6

**Published:** 2024-01-23

**Authors:** Tobias Dombrowski, Nils Pursche, Caroline Beutner, Dirk Beutner

**Affiliations:** 1 Klinik für Hals-Nasen-Ohrenheilkunde, Universitätsmedizin Göttingen, Robert-Koch-Str. 40, 37075 Göttingen, Deutschland; 2 Klinik für Dermatologie, Venerologie und Allergologie, Universitätsmedizin Göttingen, Göttingen, Deutschland

**Keywords:** Lehre, Gemeinschaftliches Lernen, Selbstbestimmtes Lernen, Medizinische Ausbildung, Online-Unterricht, Teaching, Collaborative learning, Self-directed learning, Medical education, Online education

## Abstract

**Hintergrund:**

Der Flipped Classroom (FC) ist eine mittlerweile populäre Variante des Blended Learning mit einem auf den Lernenden zentrierten, didaktischen Ansatz. Hierbei werden vorab Grundlagen eines Themas selbst erarbeitet und in der Präsenzphase gemeinsam vertieft. Getriggert durch die COVID-19-Pandemie haben sich bei ausbleibender Präsenzmöglichkeit neue Ansätze ergeben, einen volldigitalen FC umzusetzen.

**Ziel der Arbeit:**

In dieser Arbeit wurde als Pilotprojekt ein interaktiver, volldigitaler FC mit Elementen kollaborativen Lernens etabliert und die Umsetzung aufgearbeitet.

**Material und Methoden:**

Die FC-Veranstaltung zum Thema „Speicheldrüsenerkrankungen“ wurde in ein volldigitales Konzept transformiert. Nach dem Selbststudium mit digitalen Lerneinheiten oder Vorlesungsaufzeichnungen wurde die Präsenzveranstaltung online abgehalten und mit Breakout-Sessions, interaktiver Demonstration der klinischen Untersuchung inklusive Sonographie und formativem Assessment angereichert. Mit einem Fragebogen mit 27 Items erfolgt die dezidierte Evaluation der Veranstaltung.

**Ergebnisse:**

Durch die Verwendung gängiger Hard- und Software konnte die Veranstaltung auf eine stabile technische Basis gestellt werden. Insgesamt konnten die Antworten von 55 Studierenden in die Auswertung mit einbezogen werden. In den Breakout-Sessions zeigte sich eine hohe Interaktion zwischen den Teilnehmern. Die Bewertung der Veranstaltung und insbesondere des Lernfortschritts zeigten gute Ergebnisse trotz fehlender Vorbereitung der Grundlagen bei 27 % des Untersuchungskollektivs.

**Schlussfolgerung:**

Auch ein volldigitaler FC kann zu hoher Zufriedenheit führen. Für das Gelingen der Veranstaltung ist neben qualitativ hochwertiger Vorbereitung eine stabile technische Basis, ein sorgfältiges Zeitmanagement und eine geeignete Themenwahl erforderlich. Das Einbinden medizinischen Bildmaterials ist dabei in didaktisch guter Qualität möglich. Die zusätzliche Implementation von Breakout-Sessions und Voting-Tools kann dann zu einem für Dozierende und Studierende befriedigenden Lern- und Lehrerfolg in einer volldigitalen Lehrveranstaltung führen.

## Abstract (en)

**Background:**

The flipped classroom (FC) is nowadays a popular principle of blended student-centered learning. Students first prepare basic knowledge at home and subsequently meet for consolidation and a more in-depth look at a certain topic. During the COVID-19 pandemic, several groups developed approaches to also transform the characteristic FC second session into an online-only format.

**Objective:**

Herein, we present a pilot study on establishing an online-only FC format with elements of collaborative learning and its evaluation by medical students.

**Materials and methods:**

The FC design related to diseases of the salivary glands was transformed into an online-only event. After studying the basic information online, supported by self-made interactive videos and/or lecture recordings, students met in a video conference enriched by breakout sessions, interactive demonstration of the related clinical examination including ultrasound, and a formative assessment. A questionnaire with 27 items was answered by participants to evaluate the concept and the event.

**Results:**

Use of common hard- and software systems led to a technically stable video conference. A total of 55 students completed the questionnaire and were included into data analysis. During the breakout sessions, lively interaction between participants was observed. The evaluation of both the event itself and the related learning progress showed good results despite the lack of preparation beforehand in 27% of participants.

**Conclusion:**

Online-only FC designs can result in high satisfaction. High quality of online preparation, a solid technical platform, accurate time management, and a reasonable selection of topics are the main parameters contributing to successful course design. Nowadays, embedding medical imaging can be realized in appropriate quality for educational purposes. The implementation of breakout sessions and voting tools enables collaborative online learning with high levels of interaction and satisfaction for both teachers and students.

## Digitalisierung der Präsenzphase

Das Konzept des Flipped Classroom als Variante des Blended Learning hat im letzten Jahrzehnt als Veranstaltungsrahmen zunehmenden Einzug in die medizinische Lehre gefunden. Dabei findet vorab eine digitale, grundlegende Vorbereitung auf die Thematik statt, sodass in der Präsenzveranstaltung eine Vertiefung und weitere Bearbeitung eines Themas durchgeführt werden kann. So sollen insbesondere wenig interaktive Formate der medizinischen Lehre, z. B. Vorlesungen, aufgebrochen werden, um den Lernerfolg zu verbessern und die Veranstaltung auf die Lernenden zu zentrieren [14, 15]. Verschiedene Reviews und Metaanalysen zeigen dabei zwar nur moderate Effekte hinsichtlich des Lernerfolgs, die gesteigerten Lern- und Lehraktivitäten sind jedoch insgesamt hinreichend belegt [1, 4]. Probleme der Evidenz lassen sich, wie bei anderen Studien zur medizinischen Lehre, zumindest teilweise auf die Schwierigkeit geeigneter Kontrollen zurückführen [17].

In Ermangelung der Möglichkeit von Präsenzveranstaltungen sind während der COVID-19-Pandemie vielerorts neue Konzepte des Flipped Classroom entwickelt worden. Ziel dieser Neuentwicklungen war i. d. R., unter Beibehaltung der themenbezogenen, digitalen Vorbereitung thematischer Grundlagen, die Präsenzphase des Flipped Classroom ebenso digital durchzuführen [9]. Durch die Digitalisierung der Präsenzphase ist somit didaktisch und technisch eine volldigitale Struktur entstanden, welche zumindest in Deutschland in der medizinischen Lehre vorher höchst selten angewendet wurde. Deren Anwendbarkeit und Gleichwertigkeit konnte dabei vorab wissenschaftlich nicht hinreichend untersucht werden.

Auch an der Universitätsmedizin Göttingen wurde während der Pandemie eine volldigitale Veranstaltung im Flipped-Classroom-Format etabliert und wissenschaftlich begleitet. Die Vermittlung von Elementen praktischer Lerninhalte sollte hierbei in Kombination mit einem Ansatz kollaborativen Lernens eine der Präsenz gleichwertige Veranstaltung schaffen. Hier wird das innovative Konzept zusammen mit der begleitenden Analyse vorgestellt.

## Material und Methoden

An der Universitätsmedizin Göttingen ist die Lehre der HNO-Heilkunde innerhalb einer Modulstruktur im 4. klinischen Semester in das Modul „Erkrankungen der Augen, des Hals-Nasen-Ohrenbereichs, des Mundes und der Zähne“ integriert. Während des Moduls finden Seminare, klassische Vorlesungen und Unterricht am Krankenbett in der HNO-Heilkunde statt. Im Zeitraum des Projekts waren semesterabhängig 120–140 Studierende für das Modul angemeldet. Die ursprünglich als Flipped Classroom im Präsenzformat geplante Veranstaltung, als Ersatz zur Vorlesung „Speicheldrüsenerkrankungen“, wurde aufgrund der pandemiebedingten Einschränkungen in ein volldigitales Format transformiert. Die Auswahl des Themas erfolgte in hinsichtlich der didaktisch sinnvollen Möglichkeit der Integration einer Demonstration der klinischen Untersuchung und des Kopf-Hals-Ultraschalls als interaktive Elemente. Die Suprastruktur des Flipped Classroom wurde dabei beibehalten (Tab. 1):

**Table 1:** 

Phase	Inhalt	Didaktische Zuordnung	Medien
Vorbereitung	Selbststudium Thema Speicheldrüsen	Grundwissen	Digitale Lerneinheit Vorlesungsaufzeichnung
Präsenz	Klinische Untersuchung	Stoffvertiefung	Konferenzsoftware Kamera
	Sonographie		
	Digitale Gruppenarbeit	Kollaboratives Lernen, Fallbearbeitung	
	Lernerfolgskontrolle	Formatives Assessment, Voting	Konferenzsoftware
Nachbereitung	Wiederholung nach Bedarf	Stoffvertiefung	Folien der Präsenzveranstaltung
	Fragebogen	Evaluation	Digitaler Fragebogen

Als Vorbereitung auf die Präsenzveranstaltung zum Thema Speicheldrüsenerkrankungen wurde eine interaktive, digitale Lerneinheit auf der Plattform ILIAS (Integriertes Lern‑, Informations- und Arbeitskooperations-System) der Georg-August-Universität Göttingen zur Verfügung gestellt. Diese wurde auf Basis einer gekürzten Vorlesungsaufzeichnung und H5P-Interaktionen nach publiziertem Konzept erstellt [21]. Alternativ konnte eine Aufzeichnung der Vorlesung zum Thema ohne Interaktionen bearbeitet werden. Zum Semesterbeginn wurden das Konzept der Veranstaltung und die empfohlene Vorbereitung klar kommuniziert.

Die Präsenzveranstaltung erfolgte auf Basis der Konferenzsoftware Zoom und wurde in 3 Abschnitte à 15 min eingeteilt:

Zur Vertiefung des erworbenen Grundwissens wurde dabei zunächst die klinische HNO-Untersuchung mit dem Fokus auf die Untersuchung der Speicheldrüsen demonstriert. Als Ergänzung erfolgte im Anschluss ein Exkurs zur Sonographie des Halses, ebenso fokussiert auf die Kopfspeicheldrüsen. Beide Elemente wurden direkt in die Zoom-Konferenz übertragen.

Im zweiten Teil der Veranstaltung wurden die Studierenden zufällig in sog. Breakout-Sessions eingeteilt. Diese bezeichnen die vorübergehende Einteilung des Plenums in Kleingruppen zur intensiveren Bearbeitung bestimmter Aspekte des Themas. Der Administrator gab hierbei lediglich die Gruppengröße bzw. Anzahl der Gruppen (8 je Semester) vor, die Zuteilung erfolgte randomisiert. Insgesamt wurden 4 vorbereitete Patientenfälle zum Thema auf die Gruppen gleichermaßen verteilt. Diese sollten innerhalb der jeweiligen Gruppe kollaborativ bearbeitet und zur Präsentation vorbereitet werden. Die Veranstaltung wurde von 4 Dozierenden betreut, die sich im Wechsel durch die ihnen zugewiesenen Gruppen schalteten und Hilfestellung leisteten.

In der letzten Phase kehrten die Gruppen ins Plenum zurück und stellten die Fälle vor. Zudem wurden mittels der in Zoom integrierten Abstimmungsmöglichkeiten (Voting-Tool) Multiple-Choice-Fragen gestellt. Mit einer Erläuterung und Vertiefung durch die Dozierenden wurde die Veranstaltung geschlossen.

**Figure 1:**
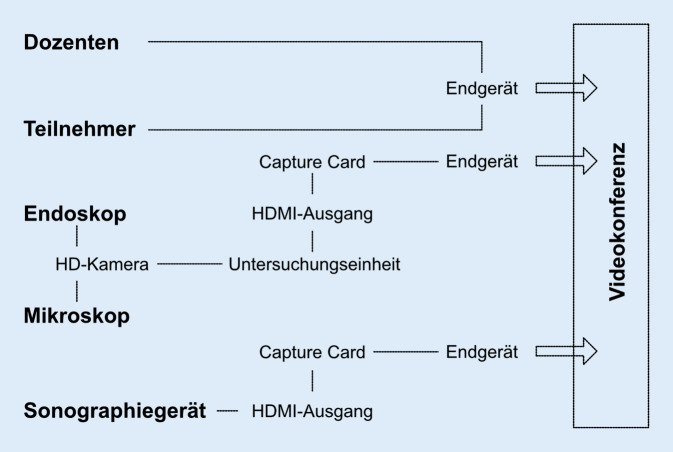

Zur digitalen Vernetzung wurde ein kostengünstiges, einfach zu installierendes Übertragungssystem rein zu Lehrzwecken ohne Zulassung für die Patientenversorgung etabliert. Basierend auf sog. Capture Cards (Elgato Game Capture HD60 S+, Fa. Corsair Gaming Inc., Milpitas/CA, USA) wurden die HDMI-Bildausgänge des Sonographiegeräts (Logiq P9, Fa. GE Healthcare GmbH, Solingen, Deutschland) und der Endoskop‑/Mikroskopkamera in Eingangssignale der High-Definition(HD)-Kamera (PES Pilot HD Pro, Fa. Happersberger Otopront GmbH, Hohenstein, Deutschland) transformiert und über das so verbundene Notebook das Bild in Zoom dargestellt (Abb. 1).

Die Evaluation erfolgte freiwillig und anonym über einen digitalen Fragebogen mit 27 Items über Microsoft Forms (Fa. Microsoft Corporation, Redmond/WA, USA). Studierende wurden über den Zweck der Datenerhebung informiert und gaben ihr Einverständnis zur weiteren Verwendung der Daten. Die Auswertung erfolgt mit Microsoft Excel (Microsoft Corporation) und R for Statistical Computing (https://www.r-project.org).

## Ergebnisse

Die Veranstaltung wurde im Wintersemester 2021/22 und Sommersemester 2022 ohne Anwesenheitspflicht mit identischen Inhalten durchgeführt. Die Teilnahmequote lag, gemessen an der Gesamtzahl der Studierenden, bei etwa 50–55 % (pro Semester jeweils 50–70 Teilnehmer). Aufgrund der etwas fluktuierenden Teilnahme während der Videokonferenz wurde diesbezüglich auf eine präzise Auswertung verzichtet und der genannte Näherungswert akzeptiert. Insgesamt 55 Studierende absolvierten eine auswertbare Evaluation, davon 16 männlich, 38 weiblich, 1 divers. Der Altersdurschnitt lag bei 25,9 Jahren.

Zunächst wurde gefragt, welche der empfohlenen Ressourcen (Vorlesungsaufzeichnung oder digitale Lerneinheit) zur Vorbereitung auf die Veranstaltung verwendet wurde (Tab. 2, *n* = 55). Beide Ressourcen wurden teilweise von 16,4 % und von 10,9 % der Studierenden ausführlicher genutzt. Dabei nutzten 5,4 % ausführlich die Vorlesungsaufzeichnung und nur teilweise die digitalen Lerneinheiten, 7,3 % ausführlich die digitalen Lerneinheiten und nur teilweise die Vorlesungsaufzeichnung. Ebenfalls 5,4 % verwendeten teilweise die digitalen Lerneinheiten und nicht die Vorlesungsaufzeichnung, 7,3 % teilweise die Vorlesungsaufzeichnung und nicht die digitalen Lerneinheiten. Ausschließlich die Vorlesungsaufzeichnung nutzten 20 %, Studierende, die ausschließlich die digitalen Lerneinheiten benutzten, gab es nicht. Zudem antworteten 27,2 % der Studierenden, sich, entgegen der vorab kommunizierten Empfehlung, gar nicht vorbereitet zu haben. Insgesamt ergaben sich so Nutzungsquoten von 45,5 % für die digitalen Lerneinheiten und 67,3 % für die Vorlesungsaufzeichnung. Unter den Studierenden, die beide Formate genutzt hatten (*n* = 28), gaben 10 Studierende an (35,7 %), dass die Vorlesungsaufzeichnung besser auf die Veranstaltung vorbereite, ebenfalls 10 Studierende (35,7 %) bevorzugten die digitalen Lerneinheiten. Die Qualität der Vorbereitung wurde für die Vorlesungsaufzeichnung in Schulnoten im Mittel mit 2,12 bewertet, für die digitalen Lerneinheiten mit 2,06.

**Table 2:** 

		Digitale Lerneinheiten		
		Ja	Teilweise	Nein
Vorlesungsaufzeichnung	Nein	0	3 (5,4 %)	15 (27,2 %)
	Teilweise	4 (7,3 %)	9 (16,4 %)	4 (7,3 %)
	Ja	6 (10,9 %)	3 (5,4 %)	11 (20 %)

**Table 3:** 

Ebene der Herausforderung	Von Lo und Hew identifizierte Herausforderungen [9]	Relevanz in dieser Studie
Studierende	Inadäquate Zeit während der Online-Veranstaltung	Nein
	Unfähigkeit, die Vorbereitung zu bewältigen	Ja
	Mangelnde Vertrautheit mit dem volldigitalen Format	Teilweise
	Negative Emotionen	Nicht analysiert
	Fehlendes Engagement von Mitstudierenden	Nein
Dozierende	Studentische technische Probleme	Nein
	Verlust echter Praxiserfahrung	Ja
	Fehlende IT-Erfahrung der Dozierenden	Nein
	Ineffektive Kommunikation	Nein
Fakultät	Erhöhter Arbeitsaufwand	Ja
	Fehlende Erfahrung mit volldigitalem Flipped Classroom	Ja
	Große Gruppen	Ja

Zu Beginn der Veranstaltung wurden die klinische HNO-Untersuchung und die Sonographie der Halsweichteile, jeweils mit Fokus auf die Speicheldrüsen, demonstriert. In Schulnoten wurde die Bildqualität der Übertragung für beide Modalitäten getrennt beurteilt. Für die Bildqualität der Sonographie ergab sich im Mittel eine Note von 1,34 (95%-Konfidenzintervall, 95%-KI: 1,03–1,66), für die klinische Untersuchung eine Note von 1,78 (95%-KI: 1,34–2,23; *p* = 0,04 im Vergleich zur Sonographie, Wilcoxon-Test). Die Nachvollziehbarkeit wurde im Mittel mit 2,03 (95%-KI: 1,55–2,51) für die klinische Untersuchung und mit 2,15 (95%-KI: 1,77–2,55) für die Sonographie bewertet. Letztere Unterschiede waren nicht signifikant (*p* = 0,41, Wilcoxon-Test).

Zum Thema Interaktivität wurde gefragt, ob die Studierenden die Möglichkeit hatten, an der Diskussion im Plenum bzw. in der Breakout-Session teilzunehmen. Hierbei antworteten 84,2 % bzw. 96,4 % mit „ja“.

**Figure 2:**
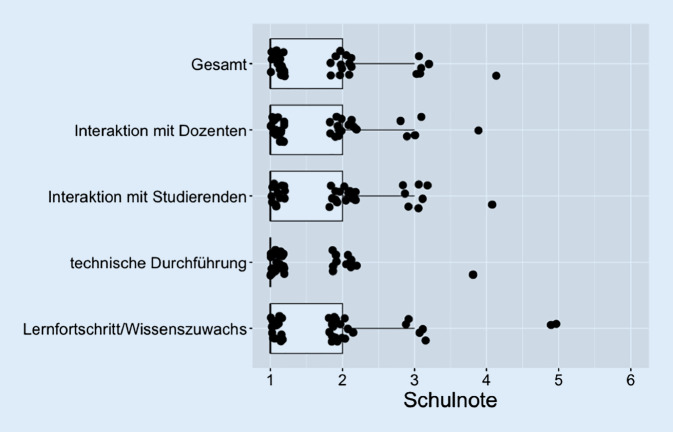

Die Ergebnisse der Bewertung wesentlicher Faktoren der Veranstaltung (Schulnoten 1–6) sind in Abb. 2 dargestellt. Hier zeigt sich in allen Items gute Bewertungen bzgl. der Interaktion mit den Dozierenden (Mittelwert 1,82; 95%-KI: 1,48–2,15) und zwischen den Studierenden (1,93; 95%-KI: 1,59–2,26), der technischen Durchführung (1,51; 1,26–1,75), des Lernfortschritts (1,71; 95%-KI: 1,46–1,96) ebenso wie der der gesamten Veranstaltung (1,82; 95%-KI: 1,48–2,16). Signifikante Unterschiede der Bewertung in sämtlichen Items zwischen den Studierenden, die sich nicht vorbereitet hatten, und den Vorbereiteten ergaben sich nicht.

Des Weiteren wurde gefragt, ob die Studierenden die reine Online-Veranstaltung als sinnvolle Ergänzung zur Präsenzlehre ansehen. Auf einer 4‑stufigen Likert-Skala befanden 42 Studierende diese Aussage als sehr zutreffend (trifft sehr zu: 76,3 %/trifft zu: 9,1 %/trifft weniger zu: 7,3 %/trifft überhaupt nicht zu 3,6 %, *n* = 53). Die Gruppengröße der Breakout-Sessions wurde überwiegend als „genau richtig“ bewertet (88,5 %, *n* = 52) und nur von wenigen Studierenden als „zu groß“ (11,5 %). Bezüglich der Dauer von 45 min gaben 34/53 (64 %) Studierenden an, dass die Dauer „genau richtig“ sei, 15 (28,3 %) empfanden die Dauer als „zu kurz“, 4 (7,5 %) als „zu lang“. In der Subgruppenanalyse befinden sich 10/15 Studierenden, welche die Dauer als „zu kurz“ bewerteten, gleichzeitig in der Gruppe, die sich nicht auf die Veranstaltung vorbereitet hatte. Umgekehrt sind 3/4 Studierenden, die die Veranstaltung als „zu lang“ empfanden, gleichzeitig in der Gruppe, welche beide Medien zur Vorbereitung genutzt haben. In ihrer persönlichen Bewertung empfanden 39/54 (72 %) die „Videokonferenz als gleichwertig zu einem Seminar“, 7 (13 %) „nicht gleichwertig zu einem Seminar“, und 7 (13 %) waren diesbezüglich unentschlossen. Auf die Frage, ob grundsätzlich das Format der Videokonferenz oder Seminare in Präsenz vorgezogen wird, entschieden sich 17/55 (30,9 %) für die Videokonferenz, 32 (58,2 %) für das Seminar in Präsenz, 6 (10,9 %) Studierende waren unentschlossen.

## Diskussion

Das Konzept des Flipped Classroom wurde in der medizinischen Ausbildung bereits über Jahre wissenschaftlich untersucht. Dabei zeigten sich auch in Metanalysen insgesamt positive Effekte hinsichtlich des Lernerfolgs, vermutlich getriggert durch verbesserte Motivation und gesteigerte Lernaktivitäten [1, 4]. Im Rahmen der COVID-19-Pandemie sind zusätzlich vermehrt Modelle des volldigitalen Flipped Classroom entwickelt worden. Lo und Hew (Tab. 3) analysierten 33 in diesem Kontext publizierte wissenschaftliche Arbeiten im Zeitraum von 2020–2021 [9]. Dabei wurde zunächst kein Widerspruch zwischen dem Konzept des Flipped Classroom und einer volldigitalen Durchführung gesehen, aber die Notwendigkeit der Auswahl geeigneter Themen betont. Zudem wurden mehrere Herausforderungen auf der Ebene der Studierenden, der Dozierenden und der Fakultät identifiziert. Eine Übersicht der identifizierten Herausforderungen und der Abgleich, ob diese auch in der vorliegenden Studie relevant waren, ist in Tab. 2 dargestellt.

Die Teilnahmequote von etwa 50–55 % erlaubt nach Ansicht der Autoren keine sicheren Rückschlüsse hinsichtlich der Motivation der Studierenden, an derartigen Formaten teilzunehmen. Die strukturierte Untersuchung dieses Faktors ist von curricularen Vorgaben und methodischen Unsicherheiten begleitet, somit aufwendig und wird daher von den Autoren als nicht praktikabel angesehen. Zusammenfassend wird die Teilnahmequote in einer subjektiven Bewertung als akzeptabel eingeschätzt. Limitierend ist in diesem Kontext, dass die Gründe für eine Nichtteilnahme in der Studie nicht erfasst werden konnten und bedingt durch die Rücklaufquote der Evaluation nur etwa die Hälfte der Teilnehmenden eingeschlossen werden konnte. Die Rücklaufquote der Evaluation ähnelt anderen Studien zum Thema, z. B. ermittelten Teichgräber et al. eine Rücklaufquote von 51,1 % [18] für die digitale Umfrage, verglichen zur historischen Kontrolle in Präsenz eine deutliche Verschlechterung (vorher 98,8 %).

Die Teilnehmeranzahl von jeweils 50–70 Teilnehmern, jeweils aufgeteilt in 8 Gruppen, wurde retrospektiv seitens der Dozierenden als günstig bewertet. Durch die vorab limitierte Anzahl an Dozierenden (4 Personen) und unklare Anzahl von Teilnehmern war im Konzept bewusst nicht die Gruppengröße der Breakout-Sessions, sondern die Anzahl der Gruppen vorgegeben. Die Betreuung von 2 Gruppen pro Dozierenden als Hilfestellung bei der Fallbearbeitung führte zur vollen Auslastung der Dozierenden. Auch von den Studierenden wurden die Gruppengrößen mit großer Mehrheit (88,5 %) als „genau richtig“ bewertet. Zudem ermöglichte die Gruppengröße von < 9 offensichtlich eine sehr gute Interaktion innerhalb der Gruppe, da 96,4 % angaben, dass sie die Möglichkeit hatten, an der Diskussion in der Gruppe teilzunehmen. Auch andere Autoren berichten über gute Erfahrungen und hohe Interaktivität von Zoom-Breakout-Sessions [3, 7] als Element kollaborativen Lernens in einer Online-Veranstaltung.

Hinsichtlich der Akzeptanz der empfohlenen Vorbereitung auf die volldigitale Veranstaltung zeigte sich, dass 27,2 % der Studierenden sich gar nicht auf die Veranstaltung vorbereitet hatten. Die Empfehlung zur Vorbereitung wurde hierbei mündlich (Einführungsveranstaltung) und schriftlich (Informationen des Studiendekanats zum Modul) an die Studierenden übermittelt, sodass diesbezügliche Unwissenheit nicht als Erklärung dienen kann. Dieser potenzielle Risikofaktor des Flipped Classroom ist allgemein in der Literatur weniger präsent. Vereinzelt vorhandene Daten zeigen jedoch ähnliche Vorbereitungsquoten [22]. Auch eine Metaanalyse von 61 Arbeiten identifizierte die mangelnde Vorbereitung als wesentliches Problem des Flipped Classroom aufseiten der Studierenden [10]. Die aufwendig produzierten digitalen Lerneinheiten wurden in dieser Studie insgesamt von 54,5 % der Studierenden nicht genutzt, während nur 32,6 % die Vorlesungsaufzeichnungen nicht nutzten. Neben der bereits an anderen Stellen vorbeschriebenen Orientierung an klassischen Formaten [21] zeigt sich in dieser Studie somit insgesamt das Bild einer eher sporadischen Vorbereitung auf die Veranstaltung. Das parallele Angebot der Vorlesungsaufzeichnung wirkt sich hier negativ hinsichtlich der Nutzung interaktiver Lerneinheiten aus, deren aus Dozierendensicht offensichtliches didaktisches Potenzial (Verbesserung der inhaltlichen Struktur der Themen, Beantwortung von Fragen, Komprimierung der Inhalte, Ergänzungen zum Thema) von über der Hälfte der Teilnehmer nicht exploriert wurde. Aus Sicht der Studierenden wurden zudem digitale Lerneinheiten und Vorlesungsaufzeichnungen gleich hinsichtlich der Vorbereitungsqualität auf die Veranstaltung bewertet. Die Autoren haben als Reaktion auf diese Ergebnisse die Vorbereitung im Rahmen von Flipped-Classroom-Veranstaltungen auf digitale Lerneinheiten reduziert, auch, um Lernziele besser fokussieren zu können. Das „unbekannte“ Format der volldigitalen Veranstaltung und die vorab unklare Prüfungsrelevanz könnten sich hier zusätzlich negativ auf die Motivation zur Vorbereitung ausgewirkt haben. Auch in der genannten Metaanalyse zum Thema volldigitaler Flipped Classroom wurde die mangelnde Vertrautheit mit dem Format als wesentliche Herausforderung auf Studierendenebene identifiziert [9]. Die unterschiedliche Vorbereitungsqualität führt in einer auf 45 min komprimierten Veranstaltung mit hoher Informationsdichte voraussichtlich zu ebenso unterschiedlichen Lerneffekten/-erfolgen. Diese These wird dadurch belegt, dass in der Gruppe 15 der Teilnehmenden, welche die Veranstaltung als „zu kurz“ empfanden, mit der Gruppe der Nichtvorbereiteten mit 66 % (10/15) deutlich überrepräsentiert ist. Dennoch wurde seitens der Studierenden der „Lernfortschritt/Wissenszuwachs“ in der Gruppe der vorbereiteten Studierenden nicht unterschiedlich bewertet verglichen zu der Gruppe ohne Vorbereitung. Zur Differenzierung zwischen subjektivem und tatsächlichem Lernerfolg könnte ein summatives Assessment am Veranstaltungsende hilfreich sein. Dazu wäre jedoch bei gegebener Informationsdichte eine Verlängerung der Veranstaltung notwendig. Andere Studien zeigen hier eine Gleichwertigkeit des volldigitalen Flipped-Classroom-Formats im Vergleich zur konventionellen Variante [18]. Grundsätzlich wird aus Studierendensicht in der vorliegenden Umfrage ein konventionelles Seminar eher bevorzugt, die volldigitale Veranstaltung aber überwiegend als eine sinnvolle Ergänzung angesehen. Diese Beobachtung deckt sich mit publizierten Evaluationsergebnissen einer volldigitalen Lehrveranstaltung von Seiwerth et al. an der HNO-Universitätsklinik in Halle [16]. Die gesamte Bewertung der Veranstaltung aus Sicht der Studierenden fällt mit einer mittleren Note von 1,82 auf einer Schulnotenskala sehr positiv aus. Durch die Implementation der Breakout-Sessions wurden in der Bewertung der Interaktion sowohl zwischen Studierenden als auch mit den Dozierenden sehr gute Ergebnisse erreicht. Dies bestätigt, dass kollaboratives Lernen auch in einem volldigitalen Umfeld erfolgreich umgesetzt werden kann. Hinweise auf die gute Umsetzbarkeit rein digitalen, kollaborativen Lernens ergaben sich bereits 2014 durch die Arbeit von McNeill et al., in der im Vergleich zur Kontrolle die kollaborative Bearbeitung von Patientenfällen zum besseren Rollenverständnis und einer besseren Lernerfahrung führten [11]. Dies wurde auf sozialen Druck und sofortiges Feedback durch andere Gruppenteilnehmer zurückgeführt. Limitierend in dieser Arbeit zeigt sich, dass zur Lernerfolgskontrolle die Daten aus der Klausur am Modulende nicht ausreichend für eine Auswertung waren. Andere Arbeiten zeigen jedoch in dieser Hinsicht keine Unterlegenheit rein digitaler Lehre [13], auch nicht in einem aktuellen Studie von Verse et al. aus der HNO-Heilkunde [20].

Herausforderungen ergaben sich im Transfer des Veranstaltungsformats bei der postpandemischen Restrukturierung der Präsenzlehre. Da sich die Studierenden im zeitlich komprimierten Modulunterricht in wechselnden Veranstaltungen innerhalb eines komplexen Stundenplans überwiegend in der Klinik bewegen, gestalteten sich sowohl die eigenständige Vorbereitung als auch die Terminierung der Online-Veranstaltung selbst als schwieriger. Ein Transfer des Formats in andere Fachabteilungen hat auch deshalb aktuell an der Universitätsmedizin Göttingen nicht stattgefunden.

Die technische Durchführung konnte durch die Nutzung gängiger Software (Zoom) und eigener Endgeräte stabil und praktikabel gehalten werden, was sich in einer guten Bewertung zeigte. Interessant ist in diesem Zusammenhang, dass die Bildqualität der klinischen Untersuchung ebenso wie der Sonographie trotz der teilweisen Nutzung von Smartphones als sehr gut bewertet wurde. Dieses Ergebnis bestätigt, dass bei Beachtung einer qualitativ guten Bildübertragung der verwendeten Hardware (in diesem Fall HD-Kamera und HD-Capture-Card) sowohl die endoskopisch/mikroskopische Untersuchung, als auch die Sonographie technisch gut vermittelt werden können, zumal Letztere noch signifikant besser bewertet wurde. Die etwas schlechter bewertete Nachvollziehbarkeit beruht nach Ansicht der Autoren zumindest teilweise auf der komprimierten Veranstaltung und der nicht auf diese Faktoren ausgerichteten Vorbereitung. Passend hierzu finden sich in der Literatur mittlerweile zahlreiche Beispiele für hybride Sonographiekurse in der prägraduierten [2, 5] und postgraduierten Lehre [8], auch für den Kopf-Hals-Ultraschall [6], welche mindestens eine Nichtunterlegenheit des Online-Formats zeigten. Dennoch finden sich hierbei keine volldigitalen Veranstaltungen. Mit der zunehmenden Implementierung von Handheld-Ultraschallgeräten könnte sich diesbezüglich jedoch ein Ansatz bieten, einen volldigitalen Ultraschallkurs für Aus- oder Weiterbildung mit dezentralen praktischen Einheiten zu entwickeln. Für die Implementierung eines volldigitalen Flipped Classroom als Rahmenstruktur für andere Anwendungen in der Weiterbildung, z. B. für einen Operationskurs ohne praktische Elemente, sind in den meisten Kliniken die technischen Voraussetzungen im Operationssaal mutmaßlich vorhanden. Herausforderungen ergeben sich jedoch auch hier in der bekannt aufwendigen Erstellung qualitativ hochwertigen digitalen Lehrmaterials als Ersatz für qualitativ hochwertige Vorträge [12].

## Ausblick

Die Komplexität eines Themas allein sollte kein Ausschlusskriterium für die Erarbeitung im volldigitalen Flipped Classroom sein [11]. Im Rahmen der Umstrukturierung des Medizinstudiums hinsichtlich des Nationalen Kompetenzbasierten Lernzielkatalogs Medizin (NKLM), ebenso wie in der Weiterbildung, ergeben sich hier möglicherweise geeignete Anwendungen des volldigitalen Formats zur Steigerung der Effektivität bzw. Zeitersparnis bei streambaren Lehrinhalten, welche Vorkenntnis erfordern und üblicherweise bisher in Präsenz in Kleingruppen absolviert werden, oder auch zur Förderung dezentralen Lernens. Es kann angenommen werden, dass im Kontext der Weiterbildung ein höheres intrinsisches Interesse am Fachmaterial vorhanden sein dürfte. Daher könnte das Format in der Weiterbildung tendenziell besser anwendbar sein, da zum einen von einer verbesserten Vorbereitungsqualität ausgegangen werden kann, zum anderen die zeitlichen Beschränkungen der curricularen Lehre nicht vorhanden sind. So könnte ein tieferer Einstieg in das jeweilige Thema ermöglicht werden. Unter genannten Einschränkungen wären beispielsweise die in einem Operations- oder Sonographiekurs üblichen Demonstrationen und theoretischen Einführungen im volldigitalen Flipped-Classroom-Format durchführbar, die Präsenz könnte dann auf die praktischen Elemente reduziert werden. Zur Gleichwertigkeit zum Präsenzformat bedarf es jedoch in der Vorbereitung hochwertiger digitaler Lehreinheiten und in der Präsenzveranstaltung selbst einer guten Struktur, insbesondere zeitlich und technisch. Dabei sollte die Zeitplanung über die Inhalte realistisch definiert werden, damit Lernziele erreicht werden können [19], zumal digitale Gruppenarbeit erwartet langsamer als in Präsenz abläuft [9]. Nach Ansicht der Autoren kann die zusätzliche Implementation von Breakout-Sessions zur Kleingruppenarbeit und Voting-Tools zur Wissensüberprüfung dann zu einem für Dozierende und Studierende befriedigenden Lern- und Lehrerfolg führen.

## Fazit für die Praxis

- Auch ein volldigitaler Flipped Classroom kann zu hoher Zufriedenheit und Interaktion führen.
- Digitale Kleingruppenarbeit in Breakout-Sessions ist dabei gut umsetzbar.
- Die Reduktion der technischen Infrastruktur auf etablierte Soft- und Hardware führte zur Minimierung technischer Probleme und der Kosten.
- Die Anwendung des Formats sollte hinsichtlich der Themen sorgfältig bedacht werden.
